# Determination of the androgen receptor status of circulating tumour cells in metastatic breast cancer patients

**DOI:** 10.1186/s12885-019-6323-8

**Published:** 2019-11-12

**Authors:** Natalia Krawczyk, Melissa Neubacher, Franziska Meier-Stiegen, Hans Neubauer, Dieter Niederacher, Eugen Ruckhäberle, Svjetlana Mohrmann, Jürgen Hoffmann, Thomas Kaleta, Malgorzata Banys-Paluchowski, Petra Reinecke, Irene Esposito, Wolfgang Janni, Tanja Fehm

**Affiliations:** 1 Department of Obstetrics and Gynaecology, University of Duesseldorf, Moorenstr. 5, 40225 Duesseldorf, Germany; 2 Department of Obstetrics and Gynaecology, Asklepios Klinik Barmbek, Rübenkamp 220, 22307 Hamburg, Germany; 3 Department of Pathology, University of Duesseldorf, Moorenstr. 5, 40225 Duesseldorf, Germany; 4 Department of Obstetrics and Gynaecology, University of Ulm, Prittwitzstraße 43, 89075 Ulm, Germany

**Keywords:** Predictive marker, Androgen receptor, Metastatic breast cancer, Circulating tumour cells

## Abstract

**Background:**

The prognostic relevance of circulating tumour cells (CTCs) in metastatic breast cancer (MBC) patients has been confirmed by several clinical trials. However, predictive blood-based biomarkers for stratification of patients for targeted therapy are still lacking. The DETECT studies explore the utility of CTC phenotype for treatment decisions in patients with HER2 negative MBC. Associated with this concept is a plethora of translational projects aiming to identify potential predictive biomarkers. The androgen receptor (AR) is expressed in over 70% of hormone receptor-positive and up-to 45% of triple-negative tumours. Studies has indicated the promising nature of AR as a new therapy target with a clinical benefit rate for anti-AR treatment in MBC patients up to 25% The aim of this analysis was the characterization of CTCs regarding the expression of the AR using immunofluorescence.

**Methods:**

MBC patients were screened for the HER2-status of CTCs in the DETECT studies. In a subset of CTC-positive patients (*n* = 67) an additional blood sample was used for immunomagnetic enrichment of CTCs using the CellSearch® Profile Kit prior to transfer of the cells onto cytospin slides. Establishment of immunofluorescence staining for the AR was performed using prostate cancer cell lines LNCaP and DU145 as positive and negative control, respectively. Staining of DAPI, pan-cytokeratin (CK) and CD45 was applied to identify nucleated epithelial cells as CTCs and to exclude leucocytes.

**Results:**

Co-staining of the AR, CK and CD45 according to the above mentioned workflow has been successfully established using cell lines with known AR expression spiked into the blood samples from healthy donors. For this translational project, samples were analysed from 67 patients participating in the DETECT studies. At least one CTC was detected in 37 out of 67 patients (56%). In 16 of these 37 patients (43%) AR-positive CTCs were detected. In eight out of 25 patients (32%) with more than one CTC, AR-positive and AR-negative CTCs were observed.

**Conclusion:**

In 43% of the analysed CTC samples from patients with MBC the AR expression has been detected. The predictive value of AR expression in CTCs remains to be evaluated in further trials.

## Background

Breast cancer (BC) is the most common malignancy in women, with almost 1.7 million new cases diagnosed per year [1]. While localized disease has become increasingly treatable, with an average 5-year survival rate of approximately 90%, metastatic breast cancer (MBC) still carries a very poor prognosis. Despite a complete removal of the tumour and adequate systemic treatment, 25–30% of primary BC patients suffer from a distant recurrence during the follow-up, making metastatic BC the second leading cause of cancer-related death among women worldwide [1–3]. Therefore, novel therapeutic targets and innovative systemic treatment approaches in MBC are still desperately required. The androgen receptor (AR) is a ligand-dependent transcription factor belonging to the nuclear steroid hormone receptor family, thus sharing several features with the oestrogen (ER) and progesterone receptors. In its unbound state, the AR is located in the cytoplasm in complex with heat shock protein 90 and other chaperone proteins. Upon ligand stimulation, the AR undergoes dimerization and translocates to the nucleus, where it regulates transcription by binding to target genes [4–6]. AR *expression has been reported* in over 70% of all primary BCs and it is more often detected in ER-positive than in ER-negative tumours. However, up to 45% of triple negative BC patients express the AR [7–14]. The role of the AR in BC has not yet been completely elucidated and seems to depend on tumour subtype. Several in vitro studies have shown a divergent effect of androgens on cell proliferation in BC cell lines [15, 16]. In the presence of ERα, the AR can either have proliferative or anti-proliferative activity, depending on the level of the co-expressed ERα and the availability of the respective ligand [17–19], Moreover, an AR-overexpression in HR-positive BC has been shown to be associated with resistance to tamoxifen, which may be reversed by an anti-androgen treatment [20]. In contrast, in HER2-positive and triple negative BC a proliferative function of the AR seems to be consistent [21]. The above indicates a strong rationale to explore AR expression as a therapeutic target in all subtypes of BC. Anti-AR treatment has recently been evaluated in two multicentre phase II studies on MBC patients showing promising results with a clinical benefit rate of up to 25% [22, 23]. The ongoing trials on anti-androgen treatment in breast cancer are summarized in Table 1. However, none of these trials included the AR-status of CTCs for stratification. Circulating tumour cells (CTCs) can be detected in approximately 40–80% of MBC patients and predict impaired clinical outcome [25]. Beyond their prognostic significance, CTCs may serve as a “liquid biopsy”, since their expression profile is assumed to most adequately reflect the phenotype of the presently dominant tumour cell population in metastatic disease. Moreover, a CTC phenotype may potentially predict the response to treatment, thereby making these cells not only a valuable source of cancer material but also a potential target for a therapeutic intervention [26]. The clinical utility of CTCs in driving treatment decisions is currently being evaluated within the DETECT studies [27]. The aim of the present substudy was to evaluate the AR status of CTCs in a cohort of MBC.

**Table 1:** Ongoing trials on anti-androgen treatment in breast cancer

Study	Status	Estimated Enrollment	Condition	Intervention	Primary Endpoint
NCT00468715 (Phase II) non-randomized	Active, not recruiting	28	AR+/HR- MBC	• Bicalutamide	CBR^a^ (observed CBR of 19% [22])
NCT01889238 (Phase II) non-randomized	Active, not recruiting	118	AR+/ triple negative ABC	• Enzalutamide	CBR (observed CBR of 25% [24])
ENDEAR trial NCT02929576 (Phase III)	withdrawn	780	Triple negative ABC	• Enzalutamide vs • Paclitaxel vs • combination	PFS
NCT02750358 (phase II) non-randomized, single agent	Active, not recruiting	200	AR+ / triple negative ESBC	• Enzalutamide	treatment discontinuation rate/ feasibility
NCT02689427 (phase IIb) non-randomized	recruiting	37	AR+ / triple negative ESBC	• Enzalutamide plus Paclitaxel in neoadjuvant setting	PCR rate
NCT02007512 (phase II) randomized	Active, not recruiting	247	HR+ HER2- ABC	• Exemestan +/− Enzalutamide	PFS
NCT02463032 (Phase II) randomized	Active, not recruiting	88	ER+/AR+ ABC	• GTx-024 (Enobosarm) • SARM • 9 vs. 18 mg.	CBR
NCT01990209 (phase II) non- randomized	Active, not recruiting	86	HR+/AR+ or triple negative /AR+ MBC	• TAK-700 (orteronel) a nonsteroidal inhibitor of CYP17A1	RR^b^ DCR^c^
NCT02067741 SAKK21/12 (Phase II) non- randomized	active, not recruiting	90	HR+/HER2- or triple negative/ AR+ ABC	• transdermal CR1447 (4-OH-testosterone)	DCR
NCT02091960 (Phase II) non- randomized	Active, not recruiting	103	HER2 + /AR + ABC	• Enzalutamide + trastuzumab	CBR

*AR* androgen receptor, *ER* oestrogen receptor, *PR* progesteron receptor, *HR* hormone receptor, *HER2* human epidermal growth factor receptor 2:, *CBR* Clinical benefit rate, ^a^ defined as proportion of patients with stability, partial response and complete response assessed by RECIST v1.1 criteria, *PFS* progression free survival, *ESBC* early stage breast cancer, *SARM* selective androgen receptor modulator, *ABC* advanced breast cancer (metastatic or locally advanced), *RR* responder rate, ^b^ defined as the percentage of complete and partial responders (CR + PR) assessed by RECIST v1.1 criteria, *DCR* disease control rate, ^c^ defined as the percentage of patients who do not exhibit progression

## Methods

### Patient material

**Fig. 1:**
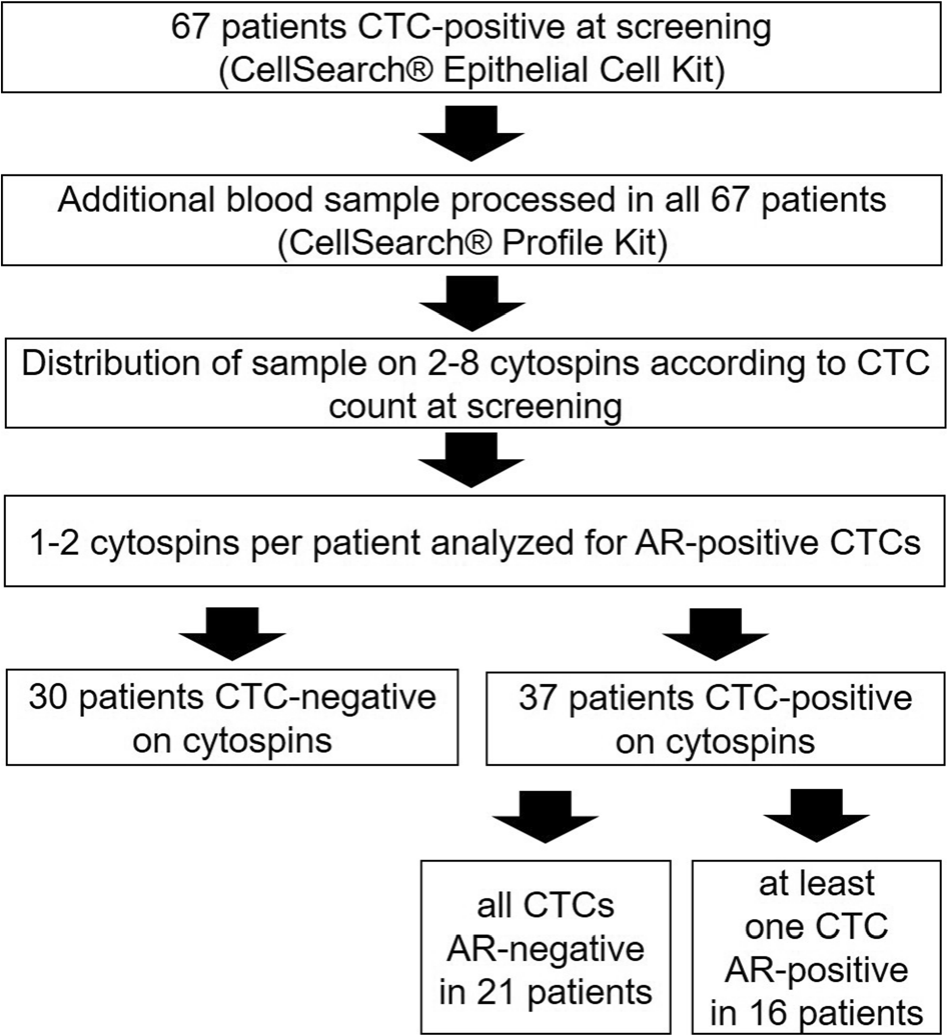
Flow chart of the trial process

Blood samples from 67 MBC patients, screened within the German DETECT III/IV trials (III: NCT01619111, IV: NCT02035813) between 2012 and 2017 for the HER2-status of CTCs, were eligible for this analysis (for more information: www.detect-studien.de). DETECT III/IV study trial is a multicenter study program for patients with HER2-negative MBC and circulating tumor cells. The main objective of this study is to evaluate the efficacy of personalized breast cancer therapy based on the presence and phenotype of CTCs. The flow chart of our substudy is presented in Fig. 1. Written informed consent was obtained from all participating patients and the study was approved by the Ethical Committee of the Eberhard Karls University of Tuebingen (responsible for DETECT III: 525/2011AMG1) and the local Ethical Committee of the Heinrich Heine University of Duesseldorf (DETECT III: MC-531; DETECT IV: MC-LKP-668).

### CTC enrichment and cytospin preparation

Blood samples were drawn into 10 ml CellSave tubes (Menarini Silicon Biosystems), maintained at room temperature and processed within 72 h after collection. The CellSearch® Epithelial Cell Kit (Menarini Silicon Biosystems) was used routinely for enrichment and enumeration of CTCs as described previously [28]. In a subset of CTC-positive patients an additional blood sample was processed using the CellSearch® Profile Kit (Menarini Silicon Biosystems) to enrich tumour cells expressing the epithelial cell adhesion molecule (EpCAM) immunomagnetically without further labelling or enumerating the cells. 10 mL of blood from the CellSave Preservative Tube was transferred into a correspondingly labelled 15 mL CELLSEARCH® Conical Centrifuge Tube with 6.5 mL of dilution buffer, consisting of phosphate buffered saline (PBS), 0.5% bovine serum albumin and 0.1% sodium azide. The sample was centrifuged at 800 x g for 10 min at room temperature and processed on the CELLTRACKS® AUTOPREP® System within 1 h. The magnetic incubation steps were performed and the vast majority of leukocytes and other blood components were depleted from the final sample. Using a ROTOFIX 32 A centrifuge (800 rpm, 2 min; Hettich GmbH & Co.KG, Tuttlingen, Germany) 400 μl of the white blood cell-depleted cell suspension were spun onto a glass slide. The slides were air-dried overnight at room temperature and stored at − 20 °C. One to two cytospins per patient was analysed for AR-positive CTCs. Control cytospins with AR-positive LNCaP cells and AR-negative Du145 cells mixed with peripheral blood mononuclear cells (PBMCs) from a healthy volunteer were similarly prepared, stored and fixed.

### Androgen receptor staining

**Fig. 2:**
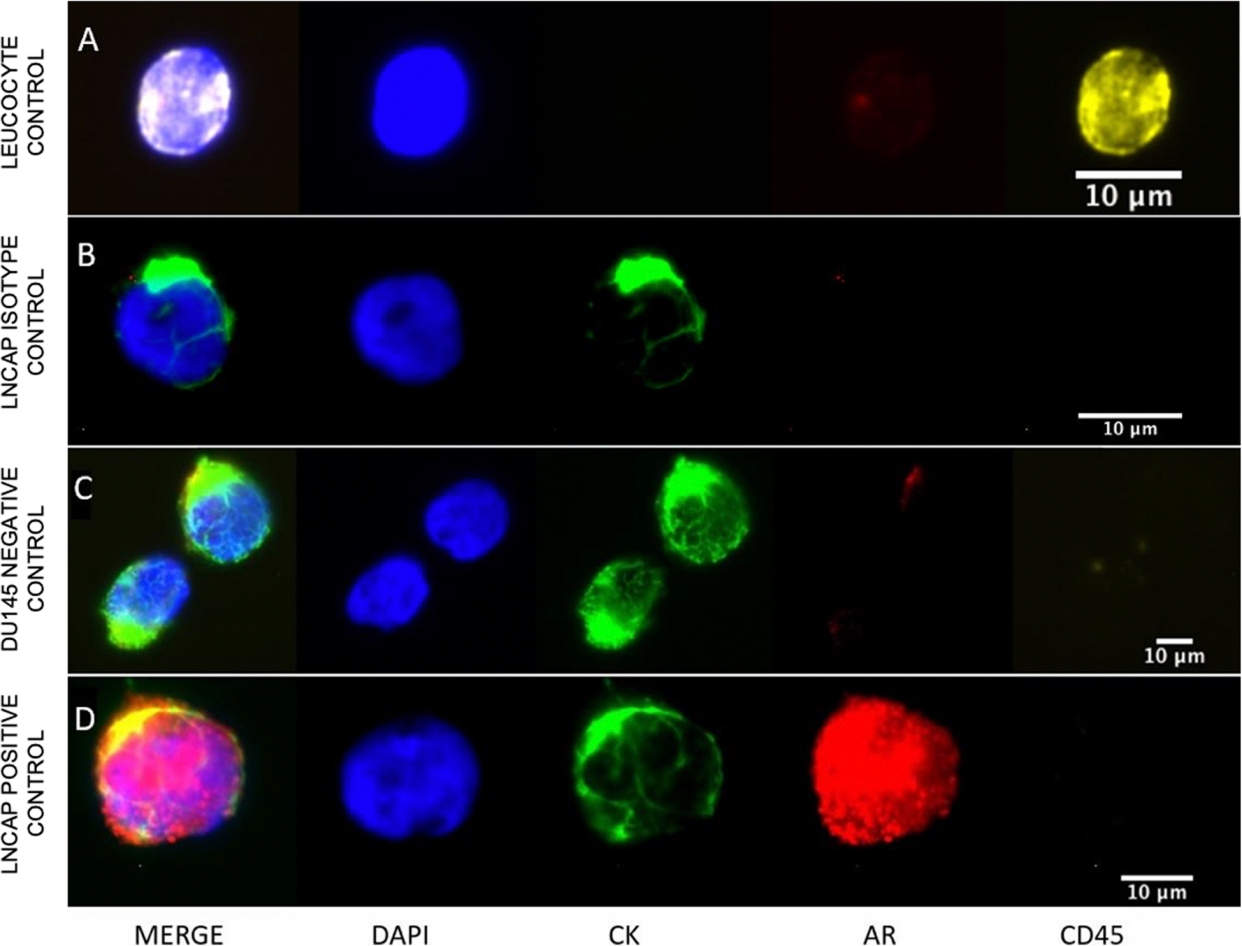
Androgen receptor (AR) control stainings (**a**) CD45 positive control staining (leucocyte) (**b**) AR isotype control staining (LNCaP) (**c**) Du145 prostate cancer cell line (negative control) (**d**) LNCaP prostate cancer cell line (positive control)

Cytospins were thawed at room temperature in a humid chamber for approximately 20 min and fixed with CellSave (Veridex, Warren, NJ, USA) for 10 min. After an initial wash step with PBS (Sigma, Munich, Germany), cells were permeabilized with PBS containing 0.1% Triton X-100 for a period of 10 min prior to blocking with Protein Block solution (DAKO, CA, USA) for another 10 min. The immunofluorescence stainings were performed using the Androgen Receptor (D6F11) XP rabbit monoclonal antibody (1:100, Cell Signaling Technologies Inc., Cambridge UK) and the pan-cytokeratin (CK) antibody (C11) directly conjugated to fluorescein isothiocyanate (FITC) (1:100, Sigma, Munich, Germany) for 60 min. Cytospins were subsequently incubated with a secondary donkey anti-rabbit antibody, labelled with Alexa Fluor 594 (1:500, Invitrogen Molecular Probes, Carlsbad, CA, USA) and an Alexa Fluor 647-conjugated CD45 antibody (35-Z6) (1:20, Santa Cruz Biotechnology, Dallas, TX, USA) for 30 min. Nuclear DNA staining was performed with 4′6-diamidino-2-phenylindole (DAPI) in mounting media (Vector Laboratories, Burlingame, CA, USA). Preparations of the prostate cancer cell line LNCaP mixed with PBMCs from a healthy volunteer served as a positive control for CK and AR staining. The AR-negative control slides of Du145/PBMC mixtures were also included with each batch of samples. CK positive, CD45 negative cells that contained an intact nucleus (DAPI positive) were identified as CTCs. Positive and negative control stainings are shown in Fig. 2.

### Statistical analysis

The chi-squared test was used to evaluate the association between CTCs and clinicopathological factors. Statistical analysis was performed by SPSS (version 25). Values of *p* < 0.05 were considered statistically significant.

## Results

### Patients` characteristics

Peripheral blood from 67 MBC patients screened for participation in the DETECT trial were eligible for this study. 55 patients (82%) had hormone receptor (HR)-positive/HER2-negative tumours, two cases (3%) had immunohistochemistry stainings indicating HR-positive/HER2-positive disease, and 10 patients (15%) had a triple negative breast cancer (TNBC). In 26 patients (40%) the blood draw was performed prior to the first line therapy for metastatic disease. The remaining 41 patients (60%) had progressive metastatic disease at blood sampling. The clinical data of the patients are summarized in Table 2.

**Table 2:** Clinical data of patients

	n N = 67	CTC positive (%)	*p*-value	AR-positive CTC (%)	p-value
Total	67	37 (55)		16 (43)	
Menopausal status			0.40		0.68
premenopausal	12	7 (58)		4 (57)	
postmenopausal	53	28 (53)		11 (39)	
unknown	2	2 (100)		1 (50)	
Line of treatment			0.75		0.30
1st	26	15 (58)		8 (53)	
≥ 2nd	41	22 (54)		8 (36)	
IHC tumour type			0.94		0.56
TNBC	10	6 (60)		2 (33)	
HR+/HER2-	55	30 (54)		14 (47)	
HR+/HER2 + ^a^	2	1 (50)		0	
Site of metastasis			0.65		0.44
bone only	14	8 (57)		4 (50)	
other site	52	28 (54)		11 (39)	
unknown	1	1 (100)		1 (100)	

^a^screening failure

### CTC detection and AR expression in CTCs

**Fig. 3:**
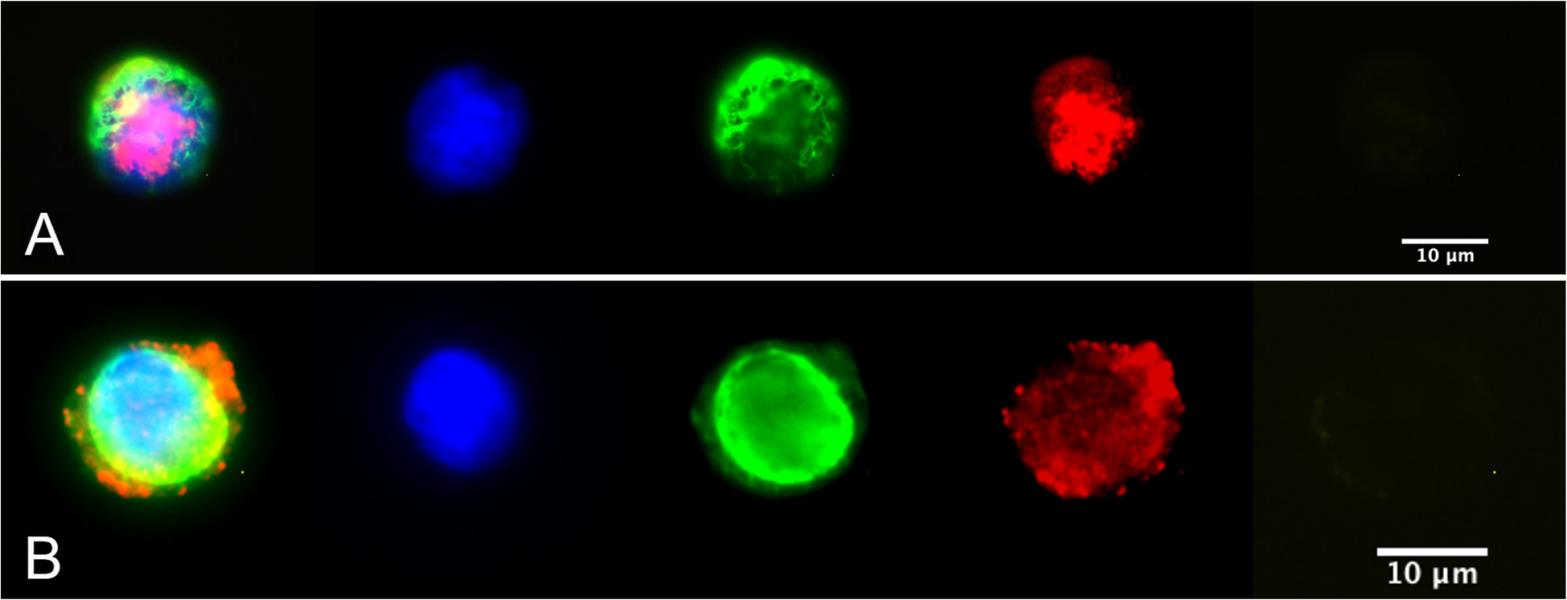
androgen receptor (AR) staining of CTCs in metastatic breast cancer patients (**a**) AR-positive nuclear staining (**b**) AR-positive cytoplasmic staining

At least one CTC was detected in 37 patients (56%). The CTC count ranged from 1 to 101 cells. In 16 out of the 37 CTC-positive patients (43%), AR-positive CTCs could be detected. The percentage of AR-positive CTCs among CTCs detected per patient ranged from 0 to 100% (mean 35.5, 95%-CI: 21.4–49.6%). In 5 out of 16 patients (31%) with AR-positive CTCs, the AR was localized in the nucleus whereas in 10 patients (62.5%) the AR signal was detected in the cytoplasm. Both nuclear and cytoplasmic localization were observed in only one patient (6.5%). Heterogenic AR localization in CTCs is depicted in Fig. 3. Among the 25 patients with more than one CTC, 14 had only AR-negative CTCs, and 3 had only AR-positive CTCs. In the remaining 8 patients (32%), AR-positive and AR-negative CTCs could be detected and the AR-positivity rate ranged from 12 to 83%. The characteristics of CTC-positive patients are demonstrated in Table 3.

**Table 3:** Characteristics of CTC-positive patients

Patient	Menopausal status	IHC tumour type	Number of previously received treatment lines^a^	Metastatic site	CTC count	AR positive CTC (%)	AR localization
1	postmenopausal	HR+ HER2-	1	bone visceral	101	84 (83)	cytoplasm/nucleus
2	premenopausal	HR+ HER2-	0	bone	13	7 (54)	cytoplasm
3	postmenopausal	HR+ HER2-	2	bone visceral	10	3 (30)	cytoplasm
4	postmenopausal	HR+ HER2-	2	bone	9	0 (0)	–
5	premenopausal	TNBC	0	bone visceral	8	1 (12)	cytoplasm
6	premenopausal	HR+ HER2-	0	bone	7	7 (100)	nucleus
7	unknown	HR+ HER2-	0	unknown	4	3 (75)	cytoplasm
8	postmenopausal	HR+ HER2-	4	bone visceral	4	3 (75)	cytoplasm
9	postmenopausal	HR+ HER2-	0	bone	3	1 (33)	cytoplasm
10	postmenopausal	HR+ HER2-	7	bone	3	3 (100)	cytoplasm
11	postmenopausal	HR+ HER2-	0	visceral	3	3 (100)	cytoplasm
12	postmenopausal	HR+ HER2-	3	bone visceral	3	0 (0)	–
13	postmenopausal	HR+ HER2-	4	bone visceral	3	0 (0)	–
14	postmenopausal	HR+ HER2-	1	bone visceral	3	0 (0)	–
15	unknown	HR+ HER2+	2	visceral	3	0 (0)	–
16	premenopausal	HR+ HER2-	0	bone lymph nodes	3	0 (0)	–
17	premenopausal	TNBC	1	bone visceral	2	1 (50)	nucleus
18	postmenopausal	HR+ HER2-	1	bone	2	0	–
19	postmenopausal	HR+ HER2-	2	bone visceral	2	0	–
20	postmenopausal	HR+ HER2-	2	bone visceral	2	0	–
21	postmenopausal	TNBC	0	visceral	2	0	–
22	postmenopausal	HR+ HER2-	2	bone lymph nodes	2	0	–
23	postmenopausal	HR+ HER2-	2	bone	2	0	–
24	postmenopausal	HR+ HER2-	1	bone visceral	2	0	–
25	premenopausal	HR+ HER2-	0	bone	2	0	–
26	postmenopausal	HR+ HER2-	1	bone visceral	1	1 (100)-	nucleus
27	postmenopausal	HR+ HER2-	0	bone visceral	1	1 (100)	nucleus
28	postmenopausal	HR+ HER2-	3	bone visceral	1	1 (100)	cytoplasm
29	postmenopausal	HR+ HER2-	0	Lymph nodes	1	1 (100)	nucleus
30	postmenopausal	HR+ HER2-	7	Bone lymph nodes	1	1 (100)	cytoplasm
31	postmenopausal	HR+ HER2-	0	visceral	1	0	–
32	postmenopausal	HR+ HER2-	0	bone visceral	1	0	–
33	premenopausal	TNBC	1	visceral	1	0	–
34	postmenopausal	HR+ HER2-	0	visceral	1	0	–
35	postmenopausal	HR+ HER2-	0	visceral	1	0	–
36	postmenopausal	TNBC	1	bone visceral	1	0	–
37	postmenopausal	TNBC	2	visceral	1	0	–

^a^for metastatic disease

## Discussion

There is growing evidence on the potential role of androgens and the AR in the pathogenesis of breast cancer. The majority of ER-positive breast cancers and up to 45% of TNBC express the AR in tumour tissue, making this biomarker an interesting therapeutic target [7–14]. AR targeting drugs, like bicalutamide or enzalutamide, are currently being evaluated in clinical trials focussing on AR-positive MBC, with favourable clinical benefit rates of up to 25% being obtained [22, 24]. However, since AR expression is not routinely assessed on BC tissue, AR expression status of MBC is mostly unknown. Archived primary tumour tissue or a direct biopsy of the metastatic lesion is required to assess the AR expression status in cases where an AR-targeted therapy is considered [22, 24]. In light of this, CTCs might serve as a ‘liquid biopsy’ and an attractive non-invasive alternative to the biopsy of a metastasis [29]. We established a triple immunofluorescence staining for the AR in CTCs and show that AR-positive CTCs can be detected in the peripheral blood of MBC patients. These findings are concordant with recently published data by Fujii et al. [30]. We used the EpCAM-based CellSearch® Profile kit for CTC detection to facilitate the identification of only tumour cells of epithelial origin. CTCs were further identified by direct visualisation of CK-positive, CD45-negative cells that contained an intact nucleus (DAPI positive). In our study, 16 out of 37 CTC-positive MBC patients (43%) also yielded AR-positive tumour cells in the peripheral blood. This positivity rate is higher than in the study by Fujii et al., where 23% AR-positive CTCs were detected in CTC-positive MBC patients [30]. This discrepancy may be due to differences in patient characteristics. The majority of patients included in our trial had HR-positive disease (57/67 patients (85%) compared to only 43/68 patients (63%) in the Fujii et al. study) and this subtype has been previously reported to be more likely to express AR [7, 14, 30]. The AR positivity rate of CTCs in our small MBC cohort amounted 43%. However, this positivity rate is lower than that reported for primary breast cancer tissue [7–14], which raises the question whether the AR status of CTCs coincides with that of the primary tumour. In the study by Fujii et al., three out of seven patients (43%) demonstrated AR-positive CTCs despite AR-negative primary tumours [30]. Phenotypic differences between the primary tumour, metastatic lesions and CTCs, with regard to other predictive factors such as ER or HER2, are a known phenomenon [28, 31–34]. Rocca et al. reported an overall concordance rate of 65% for AR expression between primary tumours and metastases [35]. Due to the lack of available tumour tissue (most of the patients were initially treated outside our centre), no comparison of the AR status between the CTCs and the corresponding tumour or metastatic lesion could be performed in our patients collective. However, as CTCs are an accepted non-invasive liquid biopsy [29], we hypothesize that the detection of AR-positive CTCs in MBC patients could be useful as a predictive factor for anti-AR treatment. The efficacy of targeting the AR in MBC patients with AR-positive CTCs need to be evaluated in further studies. Contrary to previously published analyses, we observed a heterogeneous localization of ARs in CTCs, with five out of 16 patients showing only nuclear AR staining and the majority (10 out of 16) only cytoplasmic staining. Both, nuclear and cytoplasmic staining was observed in CTCs from one patient. Previous studies defined AR positivity in the tumour tissue as a nuclear staining with a cut off value of ≥1% or ≥ 10% positive tumour cells regardless of intensity [11, 22, 36, 37]. In the analysis of the ARs in CTCs in BC patients, Fujii et al. also only counted nuclear localization of the receptor as positive [30]. However, heterogeneous subcellular localization of AR is a known phenomenon [5, 6]. Reyes et al. reported a common cytoplasmic AR localization in CTCs in metastatic castration-resistant prostate cancer patients [38]. The nuclear or cytoplasmic localization of the AR may reflect receptor activity, which mainly depends on the absence or presence of the ligand and was demonstrated to vary between cell lines [39–41]. Androgen serum levels in women are generally much lower than in men [42, 43], possibly leading to the reduced activity of the AR in breast cancer patients, which may explain the cytoplasmic localization of the receptor in some cases. On the other side, a postmenopausal status or an endocrine therapy with aromatase inhibitors increase serum levels of androgens in BC patients, which could result in AR activation and nuclear translocation [44, 45]. Interestingly, only three out of five patients presenting CTCs with exclusively nuclear AR localization were postmenopausal, compared to nine out of ten patients with a solely cytoplasmic localization. Of note is the fact that none of these five cases received an aromatase inhibitor administration at the time of blood draw. The one patient presenting with both cytoplasmic and nuclear AR localization was a postmenopausal woman treated with letrozole at the time of sample collection. Another explanation of our findings could be the genetic aberration of the AR resulting in an impaired function of the receptor [46]. Specific mutations of the AR gene can diminish or abolish its nuclear translocation abilities despite ligand binding. Mutations can also cause constitutively active, nuclear-localised AR even in the absence of the ligand [47]. Another possible reason for cytoplasmic AR localization has been proposed by Koryakina et al. [48]. In their trial on the cell cycle dependent regulation of AR in prostate cancer cell lines, a cytoplasmic localization of the receptor was shown to be characteristic of mitotic cells [48]. This might explain the relatively high rate of cytoplasmatic localized AR in our study as mitotic CTCs seem to be a common event in advanced breast cancer [49]. Whether cytoplasmic ARs can be targeted by anti-AR drugs remains to be clarified [38]. In the recent study by Kumar et al., the AR nuclear staining in BC was shown to have the highest accuracy in predicting the anti-androgen therapy response, however, with a rather modest positive predictive value of 30% [50]. In consideration of the above it is clear that the clinical relevance of heterogeneous subcellular AR localization in CTCs requires additional evaluative trials.

## Conclusion

The phenotypic characterization of CTCs, which might serve as a real-time liquid biopsy, is gaining in importance. This necessitates the identification of new predictive markers for systemic treatment in patients with MBC. The AR represents such a potential therapy target, since it is being expressed in all BC subtypes. In the present analysis we established a triple fluorescent staining of the AR in CTCs. The established robust method allowed for the direct visualization of the tumour cell and showed that AR-positive CTCs can be detected in MBC patients. AR localization in CTCs can vary and may be detected both in the nucleus and cytoplasm. Whether AR-positive CTCs are suitable to serve as a therapeutic biomarker and whether the pleiotropic AR localization has an impact on the efficacy of anti-AR agents in MBC, need to be explored in future trials.

## Data Availability

The data that support the findings of this study are available from Tanja Fehm but restrictions apply to the availability of these data, which were used under license for the current study, and so are not publicly available. Data are however available from the authors upon reasonable request and with permission of Tanja Fehm.

## Abbreviations

AR: Androgen receptor
BC: Breast cancer
CK: Cytokeratin
CTC: Circulating tumour cell
DAPI: 4′6-diamidino-2-phenylindole
EpCAM: Epithelial cell adhesion molecule
FITC: Fluorescein isothiocyanate
HR: Hormone receptor
MBC: Metastatic breast cancer
PB: Peripheral blood
PBMC: Peripheral blood mononuclear cells
TNBC: Triple negative breast cancer
